# Manifestation of lipopolysaccharide-induced tolerance in neuro-glial primary cultures of the rat afferent somatosensory system

**DOI:** 10.1007/s00011-021-01440-7

**Published:** 2021-02-13

**Authors:** Franz Nürnberger, Stephan Leisengang, Daniela Ott, Jolanta Murgott, Rüdiger Gerstberger, Christoph Rummel, Joachim Roth

**Affiliations:** Department of Veterinary Physiology and Biochemistry, Justus-Liebig-University Giessen, Frankfurter Strasse 100, 35392 Giessen, Germany

**Keywords:** LPS tolerance, Mixed neuro-glial cultures, Inflammation, Inflammatory pain, Inflammatory transcription factors, Cytokines

## Abstract

**Objective:**

Bacterial lipopolysaccharide (LPS) may contribute to the manifestation of inflammatory pain within structures of the afferent somatosensory system. LPS can induce a state of refractoriness to its own effects termed LPS tolerance. We employed primary neuro-glial cultures from rat dorsal root ganglia (DRG) and the superficial dorsal horn (SDH) of the spinal cord, mainly including the *substantia gelatinosa* to establish and characterize a model of LPS tolerance within these structures.

**Methods:**

Tolerance was induced by pre-treatment of both cultures with 1 µg/ml LPS for 18 h, followed by a short-term stimulation with a higher LPS dose (10 µg/ml for 2 h). Cultures treated with solvent were used as controls. Cells from DRG or SDH were investigated by means of RT-PCR (expression of inflammatory genes) and immunocytochemistry (translocation of inflammatory transcription factors into nuclei of cells from both cultures). Supernatants from both cultures were assayed for tumor necrosis factor-α (TNF-α) and interleukin-6 (IL-6) by highly sensitive bioassays.

**Results:**

At the mRNA-level, pre-treatment with 1 µg/ml LPS caused reduced expression of TNF-α and enhanced IL-10/TNF-α expression ratios in both cultures upon subsequent stimulation with 10 µg/ml LPS, i.e. LPS tolerance. SDH cultures further showed reduced release of TNF-α into the supernatants and attenuated TNF-α immunoreactivity in microglial cells. In the state of LPS tolerance macrophages from DRG and microglial cells from SDH showed reduced LPS-induced nuclear translocation of the inflammatory transcription factors NFκB and NF-IL6. Nuclear immunoreactivity of the IL-6-activated transcription factor STAT3 was further reduced in neurons from DRG and astrocytes from SDH in LPS tolerant cultures.

**Conclusion:**

A state of LPS tolerance can be induced in primary cultures from the afferent somatosensory system, which is characterized by a down-regulation of pro-inflammatory mediators. Thus, this model can be applied to study the effects of LPS tolerance at the cellular level, for example possible modifications of neuronal reactivity patterns upon inflammatory stimulation.

## Introduction

Nociception is the sensory response of an organism to potentially harmful stimuli. It protects the organism from injury and tissue damage [1]. Peripheral nociceptive nerve endings detect painful stimuli, e.g. strong heat, intense cold, mechanical and chemical stimuli. Their cell bodies are located in the dorsal root ganglia (DRG) [2]. From DRG neuronal information is forwarded via axons to the superficial dorsal horn (SDH) of the spinal cord including laminae I and II termed *substantia gelatinosa* [3]. Thus, DRG and SDH represent principle transfer stations for nociceptive and other somatosensory signals from the periphery to the brain.

We recently established mixed neuro-glial primary cultures from DRG [4] and from the SDH [5] and characterized the responses of both cultures to inflammatory stimulation with lipopolysaccharide (LPS). In LPS-stimulated cultures from both structures, we documented a strong up-regulation of various inflammatory genes and an enhanced nuclear translocation (i.e. activation) of inflammatory transcription factors. These changes had an impact on neuronal responses to putative neurochemical molecules involved in the generation and transfer of nociceptive signals. Capsaicin- or glutamate-evoked Ca^2+^ signals in neurons from DRG or SDH, respectively, were enhanced upon exposure to LPS [4, 5]. These modified responses might, in part, represent changes which occur during the manifestation of inflammatory or neuropathic pain [6, 7]. Modulation or attenuation of inflammatory responses within the structures of the afferent somatosensory system are therefore useful approaches to antagonize some of the pathophysiological disturbances during inflammation.

In this context, there is a specific property of LPS termed “endotoxin tolerance”. Repeated exposure of macrophages or animals to LPS causes the phenomenon of LPS tolerance [8], meaning a progressive attenuation of LPS-induced effects. Upon an initial exposure with LPS, cells or animals react with a refractory state. Within this state, the production of inflammatory mediators is diminished or absent when exposed to LPS once more [8–13]. For the first time tolerance to LPS was described when animals survived a lethal injection with LPS after pre-treatment with a sublethal dose of LPS [14]. Pathophysiological adaptions to prevent exaggerated inflammatory response to gram-negative bacteria represent important mechanisms to protect the infected host against an endotoxin shock [10]. Therefore, LPS tolerance is an essential negative feedback for disorders within gram-negative bacterial infection. In vivo LPS tolerance plays a pivotal role in reducing the mortality of sepsis, endotoxin shock and endotoxin-induced diseases [13]. In addition, LPS tolerance is linked to severe diseases including cystic fibrosis, acute coronary syndrome or cancer [11].

Owing to the complex phenotype of LPS tolerance, it was already earlier suggested that LPS tolerance does not develop from para- and autocrine regulations alone, but rather represents a characteristic cellular response, that includes different changes in receptors or changes in signaling cascades [15, 16]. Many different cellular and molecular mechanisms are discussed that might play a role in LPS tolerance: these include toll like receptor-4 (TLR-4) changes [17], negative regulating factors in the TLR-4 signaling cascade [18], micro-RNAs [19], apoptosis [20], chromatin modifications and gene re-programming of immune cells [21]. However, the exact mechanism of LPS tolerance remains elusive and may depend on the inflammatory condition.

There are just few studies, in which the LPS tolerance phenomenon was investigated in cells or structures of the nervous system. Microglia-enriched primary cultures [22] and a microglial cell line [23] showed features indicating the capability to develop LPS tolerance, i.e. diminished LPS-induced production of pro-inflammatory mediators after prolonged exposure to LPS. In contrast, stimulation of mice with LPS in vivo seemed to evoke a state of LPS tolerance in perivascular macrophages within the subfornical organ, a brain site with an incomplete blood–brain barrier, while parenchymal microglia were not involved in this effect [24]. Owing to the impact of inflammatory stimulation on the afferent somatosensory, namely the nociceptive system and based on our previous studies on primary cultures from DRG [4] and SDH [5], the central goal of this study can be summarized as follows. We aimed to establish a stimulation protocol, which can be used to characterize a putative state of LPS tolerance in mixed neuro-glial primary cultures from the afferent somatosensory system. Parameters of interest were expression profiles of cytokines with pro- and anti-inflammatory properties, stimulus-induced release of selected cytokines into the supernatants and immunocytochemical evaluation of nuclear translocation of inflammatory transcription factors in cells from LPS-treated primary cultures. We show that a state of hypo-responsiveness to an acute (short time) stimulation with LPS can be induced by pre-treatment with a lower LPS dose for a longer period. Possible consequences for the properties of an induced state of LPS tolerance within the nociceptive and thermoafferent systems are discussed.

## Materials and methods

### Primary culture from rat DRG

We used 4–6 weeks old Wistar rats from an in-house breeding to prepare primary cultures from DRG. Parent animals were obtained from Charles River WIGA (Sulzfeld, Germany). Animal care, breeding and experimental setup were performed according to the German Law on Animal Welfare, authorized by the Justus-Liebig-University of Giessen (approval number GI 577_M) and reported to the regional authority of Hessia. Room temperature was kept at 22 ± 1 °C and a relative humidity of 50 ± 5% was adjusted. Artificial lights were turned on from 7:00 to 7:00 PM.

DRG primary cultures were prepared as previously described [4, 25, 26]. Briefly, rats were killed by cervical dislocation, the vertebral column was sliced out and opened lengthwise. The spinal nerves were tracked until they merge into the dorsal root ganglia (DRG). Up to 20 DRG per rat were cut out and put into Petri dishes filled with cold, oxygenated GBSS (Gey’s Balanced Salt Solution; Sigma-Aldrich Chemie GmbH, Taufkirchen, Germany) supplemented with 0.5% d-glucose (Sigma-Aldrich Chemie GmbH). After removing residuals of the spinal nerves, the isolated DRG were transferred into oxygenated HBSS (Hanks Balanced Salt Solution, without Ca^2+^ and Mg^2+^; Biochrom GmbH, Berlin, Germany) supplemented with 20 mM HEPES (Sigma-Aldrich Chemie GmbH) at pH 7.4. Thereafter DRG were enzymatically digested using 5 mg/ml dispase II (Sigma-Aldrich Chemie GmbH) and 2.5 mg/ml collagenase (CLS II; Biochrom GmbH) dissolved in 2 ml oxygenated HBSS for 1 h at 37 °C. After enzymatical digestion, cells were dissociated mechanically and washed in HBSS containing 1 mM EDTA (Sigma-Aldrich Chemie GmbH) to inactivate the enzymes. After washing the cells twice using Neurobasal A medium supplemented with 2% B27, penicillin (100 IU/ml)/streptomycin (0.1 mg/ml) and 2 mM l-glutamine (all from: Life Technologies GmbH, Darmstadt, Germany) cells from the DRG were resuspended, cultured with a cell number of 75,000 cells/ml and plated onto poly-l-lysine (0.1 mg/ml; Biochrom GmbH) coated glass coverslips (Menzel, Braunschweig, Germany). Cells were cultured in a humidified atmosphere of 5% CO_2_ and 95% air at 37 °C. After 4 h, when the cells had attached to the glass coverslips, the medium was exchanged to remove cellular debris and the cells were used for experiments. The DRG primary culture primarily consists of neurons (10%), satellite glial cells (80%) and macrophages (1%) [4].

### Primary culture from the rat superficial dorsal horn (SDH) of the spinal cord

We used 4–6 days old Wistar rat pups from an in-house breeding (see above) to prepare primary cultures from the rat SDH of the spinal cord, predominantly the *substantia gelatinosa*. Animal care, breeding and experimental procedures were performed according to the German Law on Animal Welfare, authorized and reported as stated above (approval number GI 580_M; housing conditions: see above).

The procedure for the preparation of a mixed neuro-glial culture from the SDH was recently established and described [5]. Briefly, for preparation of SDH primary cultures, rat pups were killed by decapitation. The vertebral column was removed and cut into slices of about 1 mm. Up to 15 slices from each animal were collected in Petri dishes filled with cold, oxygenated GBSS supplemented with 0.5% d-glucose (manufacturers: see above). After removing the vertebral arch, we extracted dorsal parts of the spinal cord, which then was cleaned from surrounding spinal meninges and collected in cold, oxygenated HBSS with 20 mM HEPES at pH 7.4. The collected SDH slices were exposed to an enzyme mix containing dispase II and collagenase (see above) dissolved in oxygenated HBSS. After enzymatic digestion for 40 min, cells were dissociated mechanically and washed in HBSS containing 1 mM EDTA to stop the enzymes’ activity. After centrifugation (2 min with 2000 rpm), the supernatant was removed and cells were washed with complete medium, consisting of Neurobasal A supplemented with 2% B27, penicillin (100 U/ml)/ streptomycin (0.1 mg/ml) and 2 mM glutamine. Cells were centrifuged, re-suspended in complete medium with a cell number of 75,000 cells/ml and cultured on poly-l-lysine coated glass coverslips. Cultivation was performed in a humidified atmosphere of 5% CO_2_ and 95% air at 37 °C. The SDH primary cultures primarily consist of neurons (43%), oligodendrocytes (35%), astrocytes (13%) and microglial cells (9%) [5].

### Experimental protocol

For both, DRG and SDH primary cultures, we used the following stimulation protocol. After the cells had attached to the glass coverslips and were washed with Neurobasal A medium, half of the wells was stimulated with a moderate dose (1 µg/ml) of LPS (*Escherichia coli* serotype O111:B4; Sigma Aldrich Chemie GmbH) for 18 h, whereas the other half was treated with an equivalent volume of phosphate buffered saline (PBS, Capricorn Scientific GmbH, Ebsdorfer Grund, Germany) for 18 h as control. This dose was chosen after a series of pilot experiments, in which several LPS doses were tested. The dose of 1 µg/ml LPS fulfilled the following criteria. (1) This dose induced a profound increase in TNF-α in the supernatants of the primary cultures without any impact on the cells’ vitality, as determined by the trypan blue exclusion assay [4, 5]. (2) This dose further had a significant enhancing effect on neuronal responses to nociceptive stimuli in DRG primary cultures, as determined in Ca^2+^-imaging experiments [4].

After 18 h of incubation the microculture wells from each group pre-treated with 1 µg/ml LPS or PBS were split in half. One half of each group was stimulated with a high dose (10 µg/ml) of LPS for 2 h, the other half was treated with PBS for 2 h as control. The dose of LPS for this short-term stimulation was chosen according to previous studies, in which several doses had been tested [4, 5, 26, 27]. For short-term stimulation with LPS in mixed neuro-glial primary cultures the dose of 10 µg/ml was the lowest dose to induce a robust and significant increase of TNF-α and IL-6 release into the supernatants. This experimental procedure finally resulted in 4 different groups: PBS/PBS represent naïve cells that had no prior contact with LPS. LPS1/PBS cultures had been in contact with a moderate dose of LPS (1 µg/ml) for 18 h, followed by 2 h of treatment with PBS. PBS/LPS10 cells had been in contact with PBS for 18 h, followed by stimulation with a high dose of LPS (10 µg/ml) for 2 h. Finally, LPS1/LPS10 cultures represent the assumed tolerant group that had initial contact with the moderate dose of LPS (1 µg/ml) for 18 h followed by short-term incubation with the high dose of LPS (10 µg/ml) for 2 h.

### Real-time PCR

Real-time PCR in cells from DRG and SDH primary cultures was performed as previously described [4, 5, 26]. Solely the selected inflammatory target genes differed between DRG and SDH primary cultures. Real-time PCR was performed in four independent experiments for each stimulation group (PBS/PBS, LPS1/PBS, PBS/LPS10, LPS1/LPS10). In each of the separate experiments, 4 wells per group had to be pooled to obtain a sufficient amount of mRNA, so that 16 wells had to be used in total. After collection of the supernatants for cytokine measurements (see cytokine measurements), the cells were washed twice with PBS. Then they were lysed in 200 µl RA1-buffer, which is a component of the NucleoSpin^©^ RNA XS Kit (Macherey Nagel, Düren, Germany). The following steps were performed according to the manufacturer’s protocol. The RNA concentrations of all samples were equalized to 25 ng/µl, before performing the next steps. Reverse transcription of RNA was performed by the use of 50 U murine leukemia virus reverse transcriptase, 50 µM random hexamers and 10 mM deoxynucleoside triphosphate (dNTP) mix (Applied Biosystems, Foster City, CA, USA) in a total reaction volume of 20 µl. Quantitative real-time PCR was performed in triplicates. We used a pre-optimized primer/probe mixture (TaqMan Gene Expression Assay, Applied Biosystems, Foster City, CA, USA) and a TaqMan PCR Master Mix (Applied Biosystems) on a StepOnePlus Real-Time PCR System (Applied Biosystems). We applied a specific thermocycling protocol: polymerase activation, 50 °C for 2 min, denaturation, 95 °C for 10 min and 40 cycles of 15 s denaturation at 95 °C followed by 1 min of annealing and elongation at 60 °C.

After comparing different housekeeping gene candidates, we normalized the cDNA quantities using the reference gene β actin (Rn00667869_m1, Applied Biosystems) as endogenous reference [5, 28, 29]. The 2^−(ΔΔCt)^ method was used for relative quantification. The sample values for each gene represent x-fold difference from a control sample, given as a designated value of 1 within the same experiment. The following gene expression assays from Applied Biosystems were used: TNF-α: Rn99999017_m1, IL-6: Rn01410330_m1, IL-10: Rn99999012_m1, TLR-4: Rn00569848_m1, TRPV-1: Rn00583117_m1.

### Cytokine measurements

Highly sensitive bioassays for TNF-α and IL-6, which detect even the rather low levels of both cytokines in the supernatants of DRG and SDH primary cultures, were used to obtain information about their release into the supernatants by the cells from the four experimental groups [4, 5, 30]. The underlying principle of the TNF-α bioassay is based on the cytotoxic effect of TNF-α to the mouse fibrosarcoma cell line WEHI 164 subclone 13. Serial dilutions of samples or different concentrations of an international standard (murine TNF-α code 88/532, National Institute for Biological Standards and Control, South Mimms, UK) were incubated for 24 h in a 96 well plate that had been seeded with 50,000 actinomycin D-treated WEHI cells. The number of surviving cells after 24 h was measured by the dimethylthiazol–diphenyl tetrazolium bromide (MTT) colorimetric assay. The detection limit for the TNF-α bioassay is 6 pg/ml. The underlying principle of the IL-6 bioassay is a dose dependent growth stimulation of IL-6 on the B9 hybridoma cell line. 5000 B9 hybridoma cells were incubated for 72 h with a serial dilution of samples or with different concentrations of an international standard (human IL-6 code 89/548, National Institute for Biological Standards and Control). Evaluation was performed using the MTT assay (see above). The detection limit of the assay is 3 international units (I.U.) of IL-6.

### Immunocytochemistry

The following monoclonal antibodies or polyclonal antisera were used in DRG and SDH primary cultures for immunocytochemical identification of the cell type specific marker proteins: microtubule associated protein 2a + b for neurons (mouse AP-20 anti MAP2a + b; 1:600; Sigma-Aldrich Chemie GmbH), glial fibrillary acidic protein for satellite glial cells (DRG) or astrocytes (SDH) (rabbit anti-GFAP; 1:1000; DAKO GmbH, Hamburg, Germany; mouse anti-GFAP; 1:1000 Merck, Darmstadt, Germany) and ED1 for macrophages or microglia (mouse anti rat-ED1; 1:1000; AbD Serotec, Oxford, UK). We further used antibodies to stain TNF-α and inflammatory transcription factors: rat TNF-α (goat anti-TNF-α, 1:200, R&D Systems, Wiesbaden, Germany), signal transducer and activator of transcription 3 (STAT3, goat anti- STAT3; 1:4000; Santa Cruz, CA, USA), p65 subunit of nuclear factor kappa B (NFκB, rabbit anti-NFκB; 1:2000; Santa Cruz, CA, USA) and nuclear factor interleukin 6 (NF-IL6, rabbit anti NF-IL6; 1:4000; Santa Cruz, CA, USA). The immunocytochemical procedures were identical for DRG and SDH primary cultures.

The following protocols were used for immunocytochemical investigations. Directly after the LPS stimulation protocol (see above), cells from all four experimental groups were fixed with 4% paraformaldehyde (PFA) in phosphate buffered saline (PBS, Sigma-Aldrich Chemie GmbH), adjusted to a pH of 7.4 for 20 min. After fixation, cells were washed with PBS, 3 times for 5 min, followed by 2 h incubation in blocking buffer, containing 10% fetal calf serum (FCS, Capricorn Scientific GmbH) diluted in PBS-T containing 0.05% Triton X-100 (Sigma-Aldrich Chemie GmbH). Thereafter, the cells were incubated with the primary antibodies diluted in blocking buffer (see above) for 72 h at 4 °C in a humidified chamber. To remove unbound antibodies afterwards, the cells were washed with PBS-T 3 times for 5 min. The washing step was followed by an incubation with fluorophore-coupled secondary antisera Cy3 conjugated donkey anti-rabbit IgG (H + L) (Dianova GmbH, Hamburg, Germany) and Alexa 488 donkey anti-mouse IgG (H + L) (Life Technologies GmbH) at 1:1000 diluted in blocking buffer for 2 h. For consistency, we always chose Cy3-coupled antisera (red) for inflammatory transcription factors (NFκB, STAT3, NF-IL6) and Alexa Fluor 488-coupled antisera (green) for cell type specific marker proteins (MAP2a + b, ED1, GFAP). After three 5 min washes with PBS-T, the coverslips were embedded using a glycerol/PBS solution (Citifluor LTD, London, UK). 2,3-Diamidin-20-Phenylindol-dihydrochlorid (DAPI; Mobitec GmbH) was used for fluorescence staining of all cell nuclei. The signal intensity for detection of TNF-α was enhanced by use of a secondary biotinylated antibody (biotinylated horse anti-goat, Vector Laboratories Inc., CA, USA, 1:200 for 2 h). Therefore, the protocol included three additional steps for immunocytochemical TNF-α-staining: 15 min incubation with avidin diluted in PBS-T (1:20) at room temperature (RT), 15 min incubation with Biotin diluted in PBS-T (1:20) at RT and 60 min incubation with a Cy3 coupled Streptavidin diluted in PBS-T (1:600) [4, 5]. Cells were examined and photos were taken with a fluorescence microscope (BX-50, Olympus Optical, Hamburg, Germany) with the appropriate filter sets. Using MetaMorph microscopic imaging software (Molecular Devices, San Jose, USA) we were able to quantify the signal strength of the investigated transcription factors in the nuclei of a distinct cell type. The average intensities of the red signal (STAT3, NFκB, NF-IL6) within the region of interest (cell nuclei) were expressed in grey values [4, 5]. Mean values were compared between the 4 stimulation groups.

### Evaluation and statistics

The relative expression of inflammatory target genes in RT-PCR, the concentration of cytokines in the supernatant in specific bioassays and the mean intensities of nuclear signals of inflammatory transcription factors (immunocytochemistry) were presented as mean ± standard error of the mean (SEM). Two-way analysis of variance (ANOVA; source of variation: pre-treatment, treatment and interaction) followed by a Bonferroni post hoc test was used for statistical analysis. The statistical evaluation of the IL-10/TNF-α ratio in RT-PCR and the comparisons of IL-10 and TLR-4 expression between the PBS/PBS and LPS1/LPS10 groups were performed using an unpaired *t* test. The calculations were performed with the software package GraphPad Prism (GraphPad Software Inc., LaJolla, CA, USA). The graphical depiction of the *p* values within every experiment is displayed as follows: *** = *p* < 0.001; ** = *p* < 0.01; * = *p* < 0.05.

## Results

### Assessment of LPS tolerance in DRG and SDH primary cultures I: expression profiles of selected genes

**Fig. 1:**
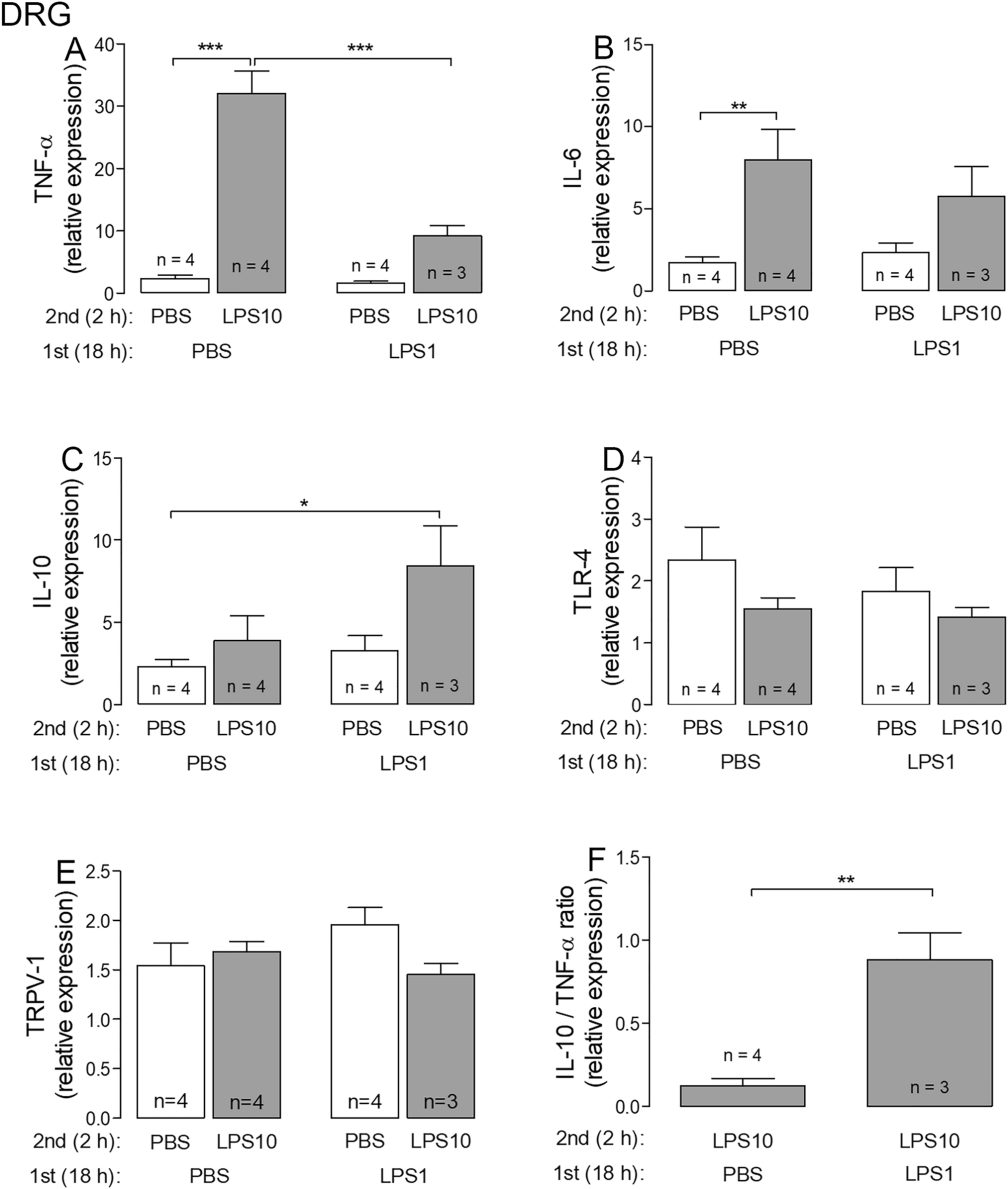
LPS tolerance affects the IL-10/TNF-α expression ratio in DRG primary cultures. LPS tolerance was induced by the cultivation of cells from rat DRG in the presence of 1 µg/ml LPS for 18 h (LPS1), followed by a short-term stimulation with 10 µg/ml LPS for 2 h (LPS10). Respective controls were treated with PBS instead of LPS. After the stimulation protocol, the cells were lysed and used for RT-PCR experiments. Each column represents the mean ± SEM of “*n*” independent experiments, in which expression of target genes was measured in triplicates. Two-way ANOVA followed by a Bonferroni posttest was used for statistical analysis. The statistical evaluation of the IL-10/TNF-α ratio was performed using an unpaired *t* test as well as the comparison of PBS/PBS vs. LPS/LPS group in the relative expression of IL-10. The graphical depiction of the *p* values was conducted as follows: *** = *p* < 0.001; ** = *p* < 0.01; * = *p* < 0.05

Owing to the limited amounts of mRNA, which was extracted from both microcultures, we had to select some specific genes, which were appropriate to assess the manifestation of LPS tolerance in our experimental model. TNF-α and IL-6 were successfully used to characterize LPS tolerance in vivo [8]. IL-10 is an important anti-inflammatory gene, which was recently suggested to be involved in the manifestation of LPS tolerance [13]. TLR-4 is the cognate receptor for LPS and its down-regulation may be a possible mechanism to induce LPS tolerance. For the DRG primary cultures we further investigated the expression of the Transient Receptor Potential Vanilloid (TRPV) 1 channel, which is the most important transducer of painful stimuli [26, 31]. Finally, we calculated the relative IL-10/TNF-α expression ratio, which has been used to characterize shifts from pro- to anti-inflammatory states or vice versa under various conditions [32, 33]. The results from these measurements and calculations are summarized in Figs. 1 (DRG) and 2 (SDH).

**Fig. 2:**
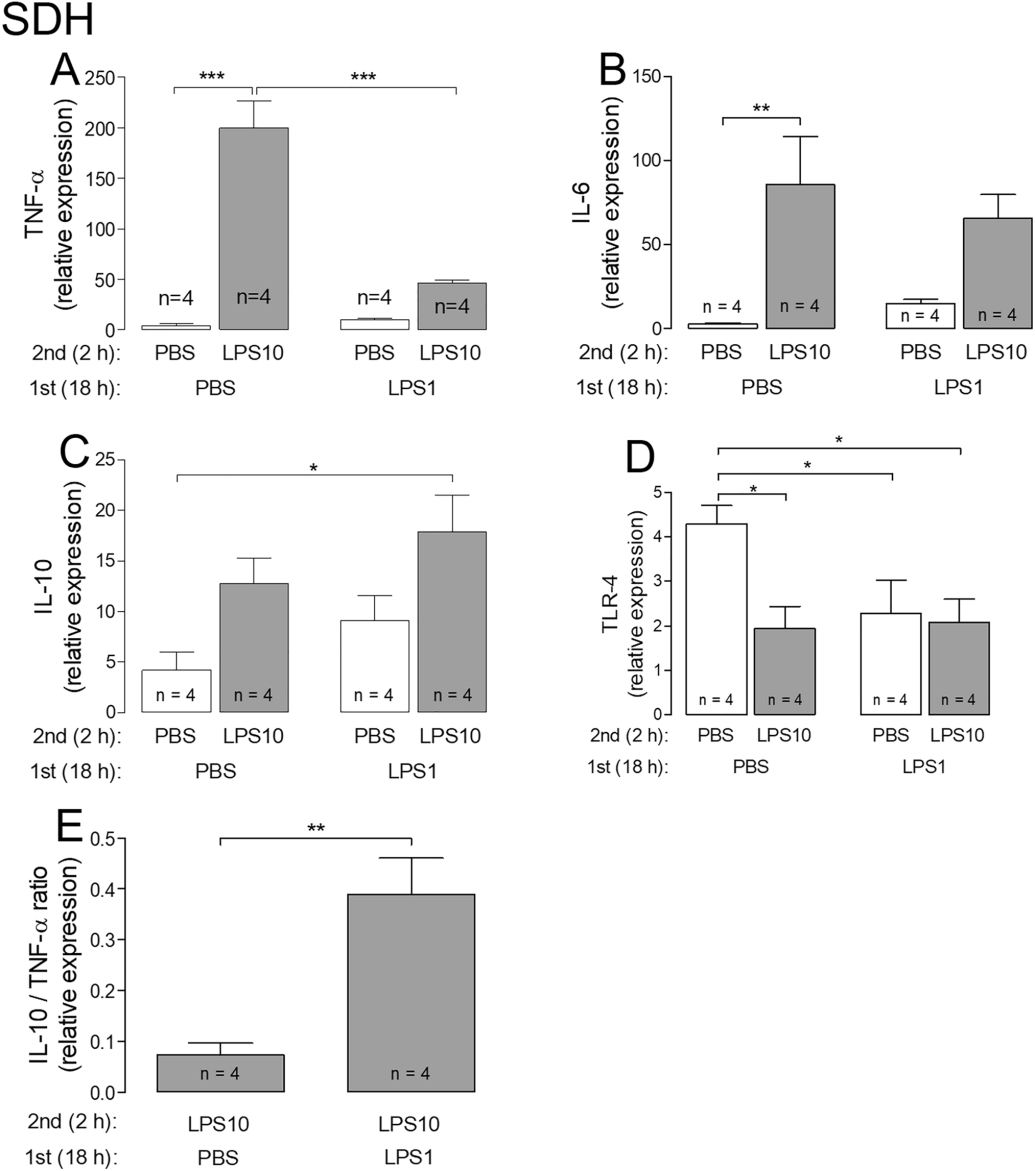
LPS tolerance affects the IL-10/TNF-α expression ratio in SDH primary cultures. LPS tolerance was induced by the cultivation of cells from the SDH of rat pups in the presence of 1 µg/ml LPS for 18 h (LPS1), followed by a short-term stimulation with 10 µg/ml LPS for 2 h (LPS10). Respective controls were treated with PBS instead of LPS. After the stimulation protocol, the cells were lysed and used for RT-PCR experiments. Each column represents the mean ± SEM of “*n*” independent experiments, in which expression of target genes was measured in triplicates. Two-way ANOVA followed by a Bonferroni posttest was used for statistical analysis. The statistical evaluation of the IL-10/TNF-α ratio was performed using an unpaired *t* test as well as the comparison of PBS/PBS vs. LPS/LPS group in the relative expression of IL-10 and TLR-4. The graphical depiction of the *p* values was conducted as follows: *** = *p* < 0.001; ** = *p* < 0.01; * = *p* < 0.05

In DRG as well as SDH primary cultures the expression profiles were similar for TNF-α, IL-6 and IL-10. Cells pre-treated with PBS for 18 h showed a pronounced increase in TNF-α expression in response to a subsequent stimulation with the high LPS dose (PBS/LPS10 group) as compared to their respective controls (PBS/PBS group). In cells from both cultures, which were pre-treated with LPS1, the LPS10 induced increase of TNF-α was no longer significant (LPS1/PBS versus LPS1/LPS10; Figs. 1a, 2a). LPS10-induced expression of TNF-α was significantly lower in the LPS1/LPS10 group compared to the PBS/LPS10 group (DRG: 32.09 ± 3.56 vs. 9.20 ± 1.65; SDH: 199.9 ± 27.1 vs. 46.1 ± 3.1; *p* < 0.001 for both comparisons, Figs. 1a, 2a). Regarding IL-6, there was just a tendency for such an effect (Figs. 1b, 2b). In contrast, pre-treatment with LPS1 for 18 h enhanced the relative expression of LPS10-stimulated IL-10 in both structures; a significant difference occurred at least between PBS/PBS- and LPS1/LPS10-treated groups (DRG: 2.32 ± 0.43 vs. 8.45 ± 2.42, *p* = 0.032, Fig. 1c; SDH: 4.19 ± 1.77 vs. 17.89 ± 3.58; *p* = 0.014, Fig. 2c). The difference between the PBS/LPS10 and the LPS1/LPS10 groups was not significant. Still, there was a tendency for an enhanced LPS10-induced expression of IL-10 in cultures pre-treated with LPS1.

The relative expression of the primary nociceptive ion-channel TRPV-1 was not affected by pre-treatment with LPS1 or short-term treatment with LPS10 in DRG primary cultures (Fig. 1e). The expression profiles for the LPS cognate receptor TLR-4, however, showed tendencies for LPS-induced changes in cultures from rat DRG (Fig. 1d) and significant changes in cells from SDH primary cultures (Fig. 2d). In all cultures with either a long-term contact with a moderate dose of LPS (LPS1, 18 h) or a short-term contact with a high LPS dose (LPS10, 2 h), or both LPS exposures, a significant reduction of TLR-4 expression as compared to naïve cells (PBS/PBS) was observed. The pre-exposure with LPS1, however, had no impact on the TLR-4 expression in LPS10-stimulated cells (PBS/LPS10 *vs*. LPS1/LPS10; n.s.).

The, perhaps, most profound result from the expression profiles of selected markers, with special regard to the manifestation of LPS tolerance could be assessed by calculation of the IL-10/TNF-α ratios (PBS/LPS10 vs. LPS1/LPS10; Figs. 1f, 2e; DRG: *p* = 0.0034; SDH: p = 0.0056). This finding provided strong evidence for the manifestation of LPS tolerance by pre-treatment with LPS1 at the mRNA level.

### Assessment of LPS tolerance in DRG and SDH primary cultures II: production of selected cytokines

**Fig. 3:**
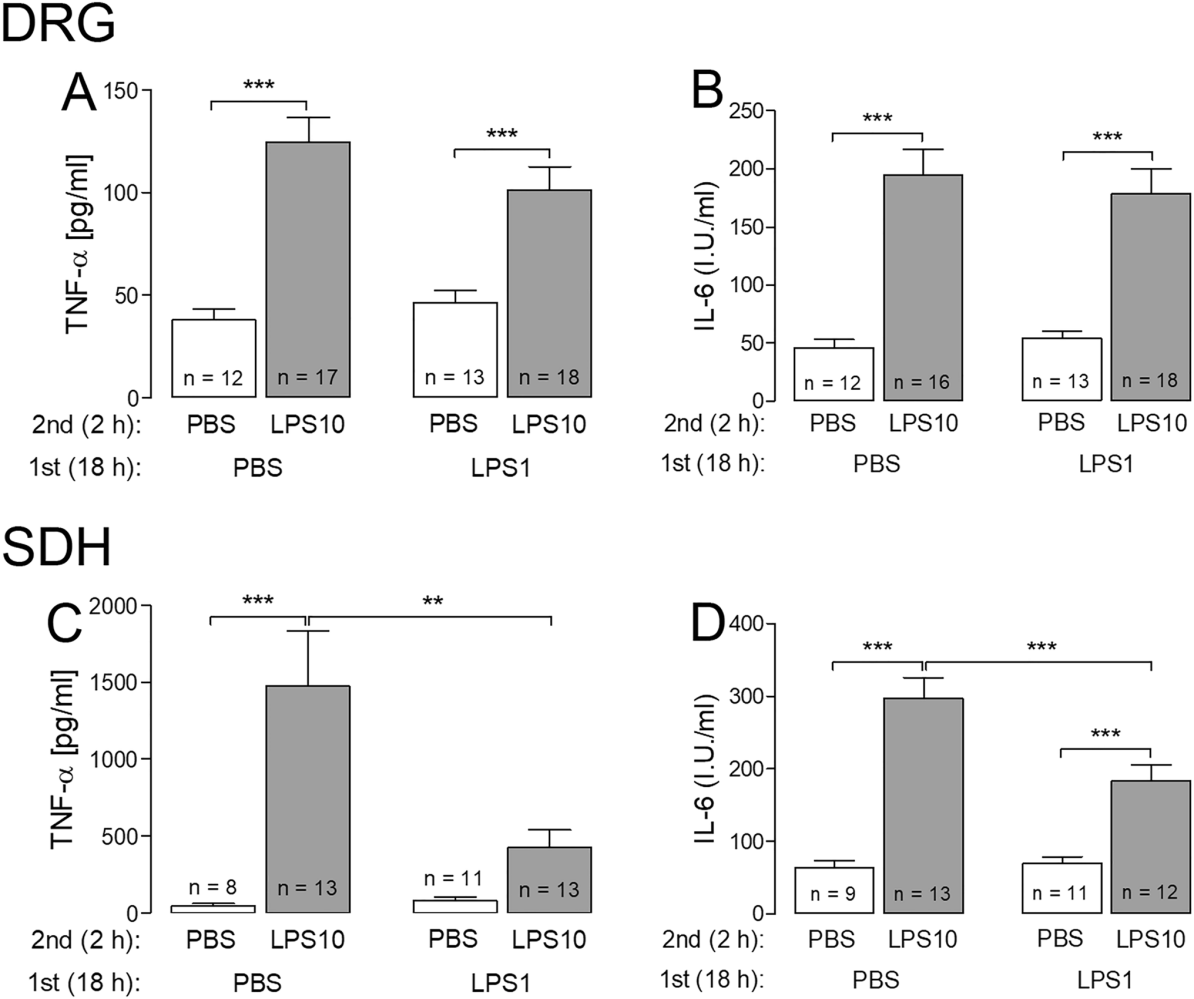
LPS tolerance affects LPS induced formation of cytokines in SDH, but not in DRG, primary cultures. LPS tolerance was induced as depicted in Figs. 1 and 2. After the stimulation protocol, the supernatants were collected for used for bioassays. Each column represents the mean ± SEM of “*n*” independent cell cultures. Statistical analysis was performed by two-way ANOVA followed by a Bonferroni post hoc test. The graphical depiction of the *p* values was conducted as follows: *** = *p* < 0.001; ** = *p* < 0.01. LPS10-induced concentrations of TNF-α and IL-6 in supernatants were significantly reduced in LPS-tolerant cultures from SDH

By the use of rather sensitive bioassays, we detected the release of the very small amounts of TNF-α and IL-6 by DRG and SDH primary cultures. The results of these measurements are summarized in Fig. 3.

In supernatants of PBS pre-treated cells from both cultures a subsequent short-term stimulation with LPS10 caused a significant increase of bioactive TNF-α and IL-6. Compared with cultures from DRG, LPS10-stimulated SDH primary cultures showed about 10-time higher levels of TNF-α and approximately twofold higher levels of IL-6. In primary cultures from the SDH the LPS10-evoked increase of TNF-α was significantly depressed when the cells were pre-incubated in the presence of LPS1 (PBS/LPS10 vs. LPS1/LPS10: 1472 ± 359 pg/ml vs. 425 ± 115 pg/ml, *p* < 0.01; Fig. 3c). Just a tendency was observed in DRG primary cultures for this response (Fig. 3a). Similar results were obtained for IL-6. The LPS10-evoked increase of IL-6 was significantly attenuated in the LPS1 pre-treated group only in cultures from the SDH (PBS/LPS10 vs. LPS1/LPS10: 297 ± 29 I.U./ml vs. 184 ± 22 I.U./ml, *p* < 0.001; Fig. 3d).

**Fig. 4:**
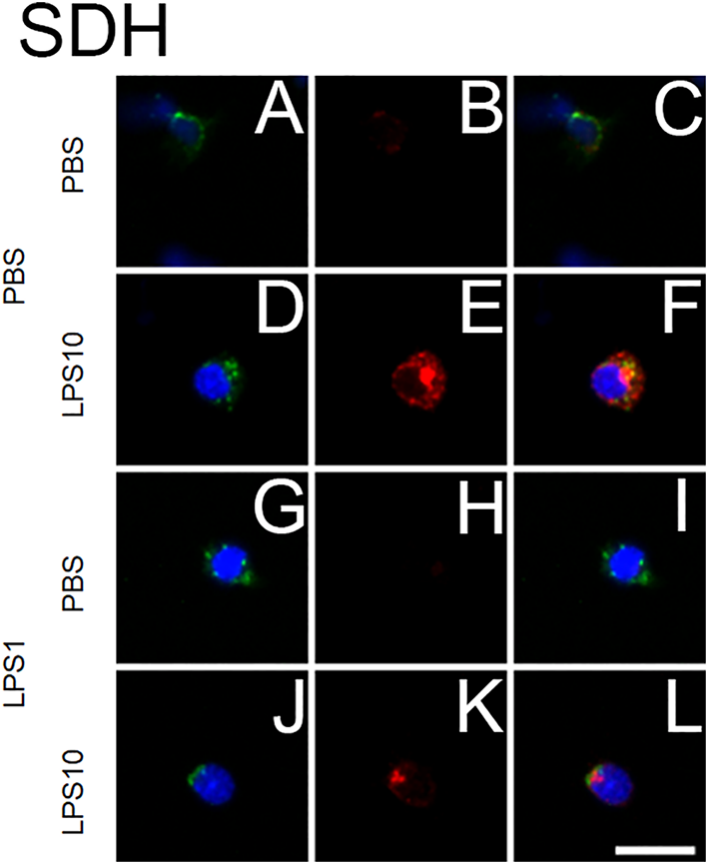
LPS tolerance affects LPS-induced immunoreactivity of TNF-α in microglial cells from SDH primary cultures. LPS tolerance was induced as depicted in Figs. 1 and 2. The four horizontal rows show examples from the four experimental groups (**a**–**c**: PBS/PBS, **d**–**f**: PBS/LPS10; **g**–**i**: LPS1/PBS; **j**–**l**: LPS1/LPS10). The first column shows ED1 immunostaining (green, marker for microglial cells) and nuclear DAPI staining (blue). The second column shows TNF-α immunostaining (red). The third column shows the overlay of the first and second columns. TNF-α immunoreactivity is predominantly seen in microglial cells of the PBS/LPS10 group, to some degree in microglial cells of the LPS1/LPS10 group and can be localized in the perinuclear Golgi complex and in vesicular organelles in the cytoplasm. The horizontal scale bar (L) represents 12.5 µm

Since pronounced effects of LPS tolerance on LPS10-induced release of TNF-α were determined in SDH primary cultures (Fig. 3), we stained SDH cells from all experimental groups with a specific antiserum against rat TNF-α (Fig. 4).

TNF-α immunoreactivity was visualized in ED1-positive microglial cells of the SDH primary culture. TNF-α signals were scarce in the PBS/PBS and in the LPS1/PBS groups. In both groups, which were stimulated with LPS10 for 2 h, clear TNF-α immunoreactivity appeared, which was more pronounced in the PBS/LPS10 compared to the LPS1/LPS10 group (Fig. 4 E, K). This observation was in line with the concentrations of TNF-α in supernatants (Fig. 3) and indicated the manifestation of LPS tolerance in the LPS1/LPS10 group. As previously reported for brain microglial cells [34], TNF-α immunoreactivity in SDH primary cultures seemed to be localized in the perinuclear Golgi complex and in cytoplasmatic vesicular organelles.

### Assessment of LPS tolerance in DRG and SDH primary cultures III: nuclear translocation of inflammatory transcription factors in microglial cells/macrophages, astrocytes and neurons

An inflammatory stimulus causes translocation of inflammation associated transcription factors (NFκB, STAT3 and NF-IL6) into the nuclei of activated cells. Their accumulation in the nucleus can be visualized immunocytochemically and quantified [4, 5]. Here we present the cellular phenotypes and transcription factors, for which modifications in the strength of nuclear signals of inflammatory transcription factors were demonstrated in our experimental model of LPS tolerance.

**Fig. 5:**
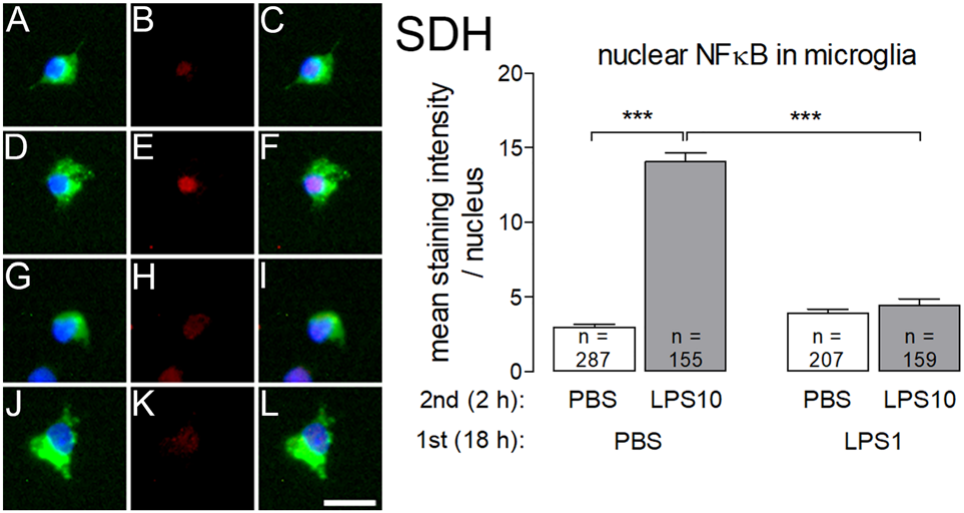
LPS tolerance affects LPS-induced nuclear NFκB immunoreactivity in microglial cells from SDH primary cultures. LPS tolerance was induced as depicted in Figs. 1 and 2. Left: The four horizontal rows are organized in the manner of Fig. 4 and show examples from the four respective experimental groups (**a**–**c**: PBS/PBS, **d**–**f**: PBS/LPS10; **g**–**i** LPS1/PBS; **j**–**l**: LPS1/LPS10). The first column shows ED1 immunostaining (green, marker for microglial cells) and nuclear DAPI staining (blue). The second column shows NFκB immunoreactivity (red). The third column shows the overlay of the first and second columns. Nuclear NFκB immunoreactivity is predominantly seen in microglial cells of the PBS/LPS10 group, to some degree in microglial cells from the other groups. Right: The intensities of nuclear NFκB-signals (red) were measured within the area of the nucleus (DAPI, blue). In microglial cells from the PBS/LPS10 (**d**–**f**) group a significantly increased NFκB immunoreactivity was observed compared to the PBS/PBS group. In LPS-tolerant cultures (LPS1/LPS10) the strength of nuclear NFκB signals was significantly reduced compared to the PBS/LPS10 group. Each column represents the mean ± SEM of n cells. Two-way ANOVA followed by a Bonferroni post hoc test was used for statistical analysis. The graphical depiction of the *p* values was conducted as follows: *** = *p* < 0.001. The horizontal scale bar (L) represents 12.5 µm

LPS-induced nuclear NFκB immunoreactivity occurred in microglial cells from SDH and in macrophages from DRG primary cultures. In the macrophages from DRG, immunoreactivity was reduced by induction of LPS tolerance, but did not exclusively accumulate in the cell nuclei upon LPS stimulation. Therefore, quantification of nuclear NFκB immunoreactivity was only performed for SDH-derived microglial cells (Fig. 5).

The pronounced LPS10-induced increase of nuclear NFκB immunoreactivity was completely abolished by LPS1 pre-treatment (PBS/LPS10 vs. LPS1/LPS10: *p* < 0.001).

**Fig. 6:**
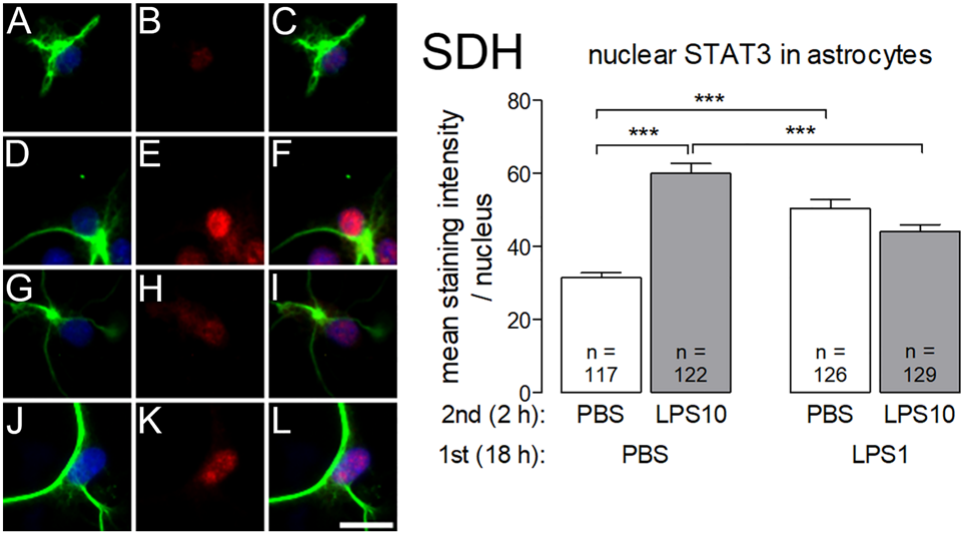
LPS tolerance affects LPS-induced nuclear STAT3 immunoreactivity in astrocytes from SDH primary cultures. LPS tolerance was induced as depicted in Figs. 1 and 2. Left: The four horizontal rows are organized in the manner of Fig. 4 and show examples from the four respective experimental groups (**a**–**c**: PBS/PBS, **d**–**f**: PBS/LPS10; **g**–**i**: LPS1/PBS; **j**–**l**: LPS1/LPS10). The first vertical column shows GFAP immunostaining (green, marker for astrocytes) and nuclear DAPI staining (blue). The second vertical column shows STAT3 immunoreactivity (red). The third vertical column shows the overlay of the first and second columns. Nuclear STAT3 immunoreactivity is predominantly seen in astrocytes of the PBS/LPS10 group, to some degree in astrocytes of the other groups. Right: The intensities of nuclear STAT3-signals (red) were measured within the area of the nucleus (DAPI, blue). In astrocytes from the PBS/LPS10 (**d**–**f**) group a significantly increased STAT3-immunreactivity was observed, compared to the LPS1/LPS10 group. Each column represents the mean ± SEM of n cells. Two-way ANOVA followed by a Bonferroni post hoc test was used for statistical analysis. The graphical depiction of the *p* values was conducted as follows: *** = *p* < 0.001. The horizontal scale bar (L) represents 12.5 µm

Nuclear activation of the transcription factor STAT3 was detected in GFAP-positive astrocytes from SDH primary cultures (Fig. 6), but not in satellite glial cells from DRG. Significantly enhanced nuclear STAT3 immunoreactivity was detected in astrocytes from the PBS/LPS10 group. This effect was blunted by the pretreatment with the low LPS dose in the LPS1/LPS10 group (PBS/LPS10 vs. LPS1/LPS10: *p* < 0.001), while basal STAT3-signal activity was higher in LPS1 pre-treated compared to PBS pre-treated astrocytes.

**Fig. 7:**
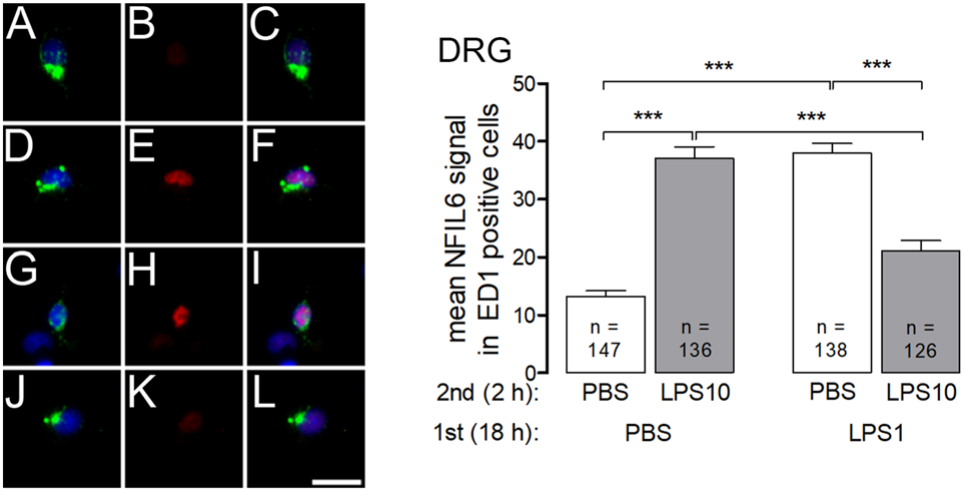
LPS tolerance and LPS pre-treatment affect LPS-induced nuclear NF-IL6 immunoreactivity in macrophages from DRG primary cultures. LPS tolerance was induced as depicted in Figs. 1 and 2. Left: The four horizontal rows (left) are organized like in Fig. 4 and show examples from the four respective experimental groups (**a**–**c**: PBS/PBS, **d**–**f**: PBS/LPS10; **g**–**i**: LPS1/PBS; **j**–**l**: LPS1/LPS10). The first column shows ED1 immunostaining (green, marker for macrophages) and nuclear DAPI staining (blue). The second column shows NF-IL6 immunoreactivity (red). The third column shows the overlay of the first and second columns. Nuclear NF-IL6 immunoreactivity is predominantly seen in macrophages of the PBS/LPS10 and of the LPS1/PBS groups, to some degree in macrophages from the other groups. Right: The intensities of nuclear NF-IL6-signals (red) were measured within the area of the nucleus (DAPI, blue). In macrophages from the PBS/LPS10 (**d**–**f**) group a significantly increased nuclear NF-IL6 immune reactivity was observed, compared to the PBS/PBS group. In LPS-tolerant cultures (LPS1/LPS10), the strength of nuclear NFκB signals was significantly reduced as compared to the PBS/LPS10 group. Pre-treatment with LPS followed caused a long-lasting increase nuclear NF-IL6 immunoreactivity, which was not reduced by short-term exposure to PBS. Each column represents the mean ± SEM of n cells. Two-way ANOVA followed by a Bonferroni posttest was used for statistical analysis. The graphical depiction of the *p* values was conducted as follows: *** = *p* < 0.001. The horizontal scale bar (L) represents 12.5 µm

**Fig. 8:**
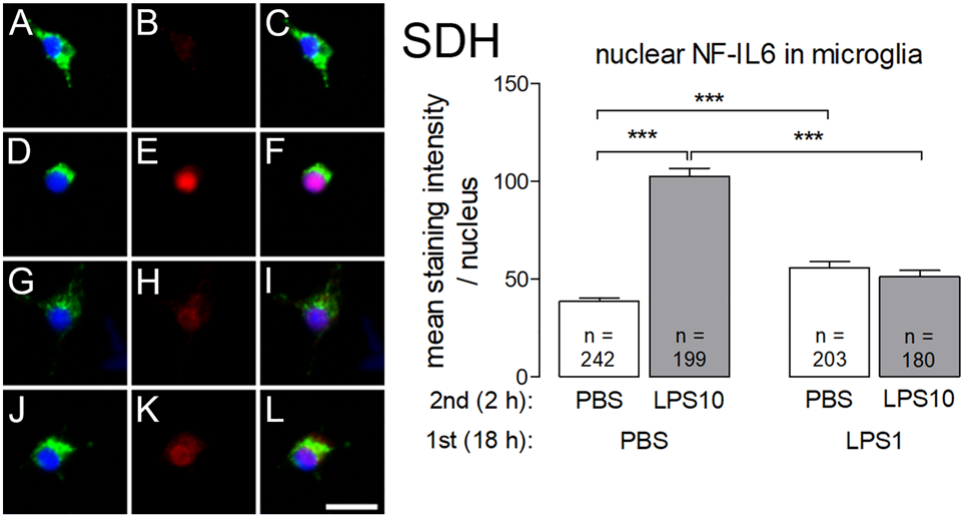
LPS tolerance affects LPS-induced nuclear NF-IL6 immunoreactivity in microglial cells from SDH primary cultures. LPS tolerance was induced as depicted in Figs. 1 and 2. Left: The four horizontal rows are organized in the manner of Fig. 4 and show examples from the four respective experimental groups (**a**–**c**: PBS/PBS, **d**–**f**: PBS/LPS10; **g**–**i**: LPS1/PBS; **j**–**l**: LPS1/LPS10). The first column shows ED1 immunostaining (green, marker for microglial cells) and nuclear DAPI staining (blue). The second column shows NF-IL6 immunoreactivity (red). The third vertical column shows the overlay of the first and second columns. Nuclear NF-IL6 immunoreactivity is predominantly seen in microglial cells of the PBS/LPS10 group, to some degree in microglial cells from the other groups. Right: The intensities of nuclear NF-IL6-signals (red) were measured within the area of the nucleus (DAPI, blue). In microglial cells from the PBS/LPS10 (**d**–**f**) group a significantly increased NF-IL6 immunoreactivity was observed compared to the PBS/PBS group. In LPS-tolerant cultures (LPS1/LPS10) the strength of nuclear NFκB signals was significantly reduced compared to the PBS/LPS10 group. Nuclear NF-IL6 immunoreactivity in the LPS1/PBS group was higher compared to the PBS/PBS group. Each column represents the mean ± SEM of n cells. Two-way ANOVA followed by a Bonferroni post hoc test was used for statistical analysis. The graphical depiction of the *p* values was conducted as follows: *** = *p* < 0.001. The horizontal scale bar (L) represents 12.5 µm

Nuclear immunoreactivity of the transcription factor NF-IL6 could be quantified in ED1-positive macrophages from the DRG (Fig. 7) and in ED1-positive microglial cells from the SDH primary cultures (Fig. 8).

There was a discrepancy with regard to the strength of LPS-induced nuclear NF-IL6 signals between the DRG macrophages (Fig. 7) and SDH microglial cells (Fig. 8). In macrophages from the DRG as well as in microglial cells from the SDH, which were pre-treated with PBS, subsequent short term stimulation with LPS10 caused a significant increase in nuclear NF-IL6 immunoreactivity (PBS/PBS vs. PBS/LPS10: *p* < 0.001 for both cultures). Pre-treatment with LPS1 caused an attenuation of LPS10-induced nuclear NF-IL6 signals in ED1 positive cells. However, the pre-treatment with LPS1 for 18 h obviously caused a long-term activation of NF-IL6 in DRG macrophages (Fig. 7), which remained manifest in the LPS1/PBS group, while this transcription factor showed a significant decrease in nuclear signals by the second stimulus with the high LPS dose (DRG: LPS1/PBS vs. LPS1/LPS10: *p* < 0.001). Such a long-term activation of NF-IL6 by pre-treatment with 1 µg/ml LPS was also observed in microglial cells from SDH primary cultures (Fig. 8: PBS/PBS vs. LPS1/PBS: *p* < 0.001), although to a smaller degree.

**Fig. 9:**
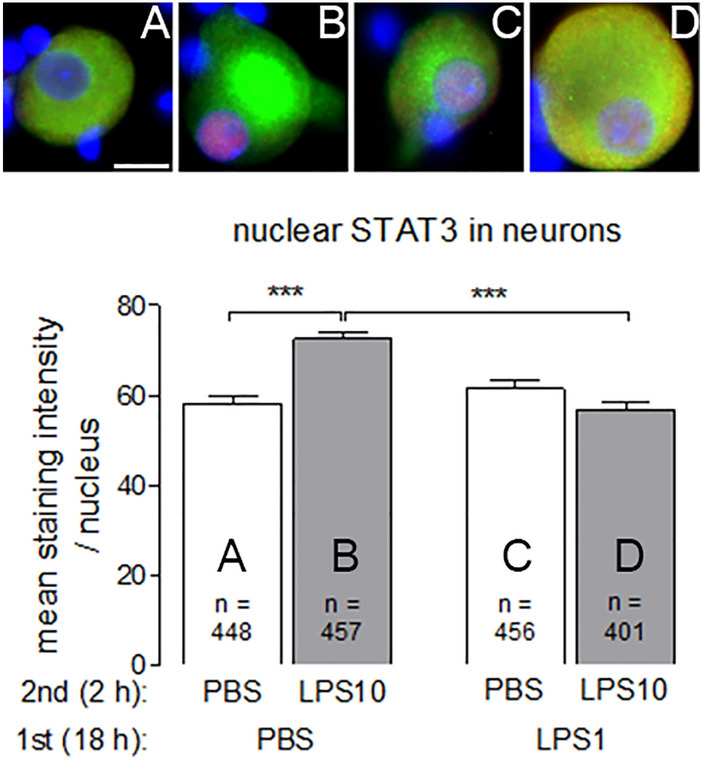
LPS tolerance affects LPS-induced nuclear STAT3 immunoreactivity in neurons from DRG primary cultures. LPS tolerance was induced in DRG primary cultures as depicted in Figs. 1 and 2. Top: Examples of neurons from the four experimental groups (**a** PBS/PBS, **b** PBS/LPS10; **c** LPS1/PBS; **d** LPS1/LPS10). MAP immunostaining (green, marker for neurons) and nuclear DAPI staining (blue) were combined with STAT3 immunostaining (red). Nuclear STAT3 immunoreactivity is moderately increased in neurons of the PBS/LPS10 group compared to the other groups. Bottom: The intensities of nuclear STAT3-signals (red) were measured within the area of the nucleus (DAPI, blue). In neurons from the PBS/LPS10 (**b**) group a significantly increased STAT3-immunreactivity was observed, compared to the PBS/PBS group and the LPS1/LPS10 group. Each column represents the mean ± SEM of n cells. Two-way ANOVA followed by a Bonferroni post hoc test was used for statistical analysis. The graphical depiction of the *p* values was conducted as follows: *** = *p* < 0.001. The horizontal scale bar (**a**) represents 12.5 µm

**Fig. 10:**
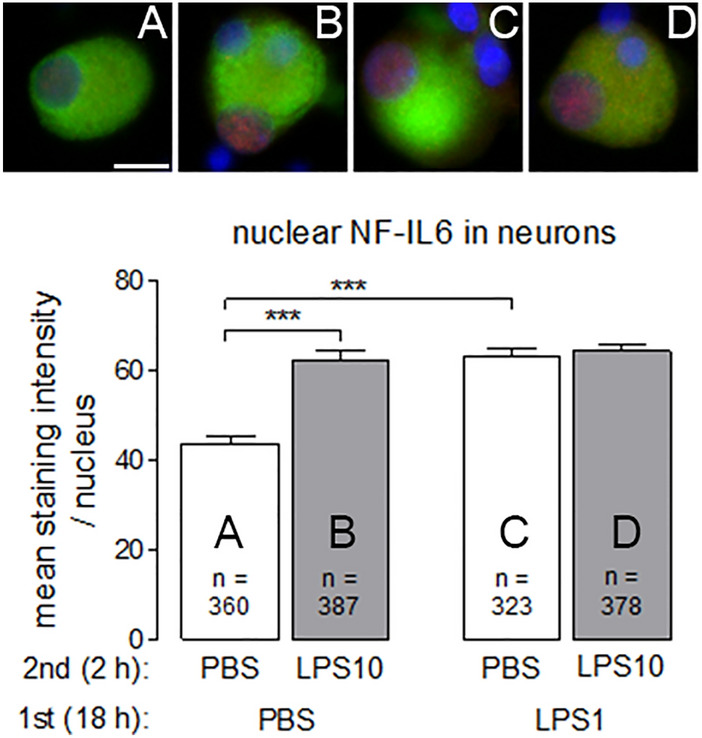
LPS increases nuclear NF-IL6 immunoreactivity in neurons from DRG primary cultures, which is not affected by LPS tolerance. LPS tolerance was induced in DRG primary cultures as depicted in Figs. 1 and 2. Top: Examples of neurons from the four experimental groups (**a**: PBS/PBS, **b**: PBS/LPS10; **c**: LPS1/PBS; **d**: LPS1/LPS10). MAP immunostaining (green, marker for neurons) and nuclear DAPI staining (blue) were combined with NF-IL6 immunostaining (red). Nuclear NF-IL6 immunoreactivity is moderately increased in neurons from all groups, which had contact with LPS1, LPS10 or both. Bottom: The intensities of nuclear NF-IL6-signals (red) were measured within the area of the nucleus (DAPI, blue). In neurons from the PBS/LPS10 (**b**) group a significantly increased nuclear NF-IL6-immunreactivity was observed as compared to the PBS/PBS group (**a**) and the LPS1/PBS (**c**) showed higher nuclear NF-IL6 immunoreactivity as compared to the PBS/PBS group (**a**). Each column represents the mean ± SEM of n cells. Two-way ANOVA followed by a Bonferroni post hoc test was used for statistical analysis. The graphical depiction of the *p* values was conducted as follows: *** = *p* < 0.001. The horizontal scale bar (L) represents 12.5 µm

An influence of inflammatory stimulation with LPS on inflammatory transcription factors in neurons was observed for STAT3 and NF-IL6 in nuclei of neurons from DRG, but not of neurons from the SDH. These data are summarized in Figs. 9 and 10.

Nuclear STAT3 immunoreactivity was detected in DRG neurons from all of the four treatment groups. In naïve neurons pre-treated with PBS followed by short-term stimulation with LPS10 caused a significant increase in the strength of nuclear STAT3 immunoreactivity (Fig. 9: PBS/PBS vs. PBS/LPS10: *p* < 0.001). This effect was abolished by pre-treatment with LPS1 (Fig. 9: PBS/LPS10 vs. LPS1/LPS10: *p* < 0.001). Nuclear NF-IL6 immunoreactivity was increased in neurons from DRG primary cultures by all kinds of LPS treatments (long-term stimulation with LPS1 or short-term stimulation with LPS10, Fig. 10), which is indicative of a general long-term activation (nuclear accumulation) of this transcription factor without manifestation of obvious tolerance effects.

## Discussion

The central goal of this study was to investigate, whether the phenomenon of “endotoxin tolerance” or “LPS tolerance”, the reduced responsiveness to endotoxin after a previous confrontation with LPS, is inducible in neuro-glial structures of the afferent somatosensory system. For this purpose, we used mixed neuro-glial primary cultures from the rat DRG and SDH. We induced tolerance to LPS using a long-term stimulation with a moderate LPS dose (1 µg/ml LPS for 18 h, LPS1) followed by a subsequent challenge with a short term stimulation with a high LPS dose (10 µg/ml for 2 h, LPS10) and investigated whether this group shows altered LPS10-induced inflammatory gene expression, altered cytokine formation and altered activation of inflammatory transcription factors. Indeed, our data suggest the manifestation of LPS tolerance within both investigated structures.

### Expression profiles of inflammatory genes and formation of cytokines

At the mRNA and the protein levels, the most pronounced tolerance effect was determined for TNF-α. In both investigated neuro-glial structures we observed a highly significant reduction of LPS10-induced expression of TNF-α after pre-treatment with LPS1. This result is in line with studies on peripheral macrophages demonstrating that a drastic reduction of TNF-α induction is a key read-out for endotoxin tolerance under in vivo or in vitro conditions [9–12, 35, 36]. Indeed, the LPS-tolerance effect in our experiments was much more pronounced for TNF-α compared to IL-6 (Figs. 1, 2 and 3). Other studies suggested that LPS tolerance is not simply a reduction of TNF-α expression and formation, but rather a re-programming of macrophages from a pro- to an anti-inflammatory state [9, 11]. The operation of such mechanisms in our DRG and SDH primary cultures is indicated by a tendency for increased expression of IL-10 in the LPS1/LPS10 compared to the PBS/LPS10 group (Figs. 1 and 2). Even better evidence for such a switch from a pro- to an anti-inflammatory state in LPS tolerance is provided by the calculations of the relative IL-10/TNF-α expression ratios, which were significantly enhanced in DRG and SDH primary cultures (Figs. 1f and 2e). These calculations are in line with the data from other publications, which show an up-regulation of IL-10 in a state of LPS tolerance [12, 13, 37, 38]. The importance for IL-10 in this context is evident in IL-10-deficient mice, which lose the ability to regulate LPS-induced expression of tolerance related molecules [39]. In further studies, high IL-10/TNF-α ratios were associated with a better outcome in children with malaria infections [40], with a reduced infection susceptibility in severe burn injury patients [33] or with improved outcome in experimentally induced myocardial infarction [32]. Transferred to our measurements, these studies suggest that a state of LPS tolerance, accompanied by increased IL-10/TNF-α expression ratios, could have some beneficial or even protective value for a subsequent tissue damage or confrontation with another severe inflammatory stimulus within the afferent somatosensory system.

We also measured the expression of TLR-4, which is the cognate receptor for LPS in monocytes / macrophages and also in cells from neuro-glial structures within the CNS [41–43]. Some authors reported that decreased TLR-4 expression in peritoneal macrophages [17] or changes within the TLR-4-evoked signaling cascade [44, 45] are critical for the manifestation of LPS tolerance. Investigations of TLR-4 expression under conditions of LPS tolerance have not been performed in structures from the peripheral or central nervous system, according to our knowledge. Interestingly, our data on TLR-4 expression partially support the view of some contribution but not a critical role in LPS tolerance. Namely, in primary cultures from the SDH all cells, which were confronted with LPS, irrespective of dose and duration, show a significant down-regulation of TLR-4 expression when compared with naïve cultures (Fig. 2d). A non-significant tendency for this effect can also be seen in primary cultures from DRG (Fig. 1d). This effect of a reduced TLR-4 expression could, however, be rather due to the exposure with an agonist, e.g. LPS alone and a contribution of this effect on the manifestation of LPS tolerance is questionable since all groups with the exception of naïve cells showed similar responses (PBS/LPS10, PBS/LPS1, LPS1/LPS10).

### Nuclear translocation of inflammatory transcription factors

Microglial cells from the SDH and macrophages from the DRG seem to be the major targets for LPS and respond to this stimulus with formation of cytokines, namely TNF-α (Fig. 4). TLR-4-mediated production of TNF-α, IL-6 and other cytokines has an impact on cellular elements within the afferent somatosensory system. This impact can be assessed by the accumulation of several inflammatory transcription factors in nuclei of LPS- or cytokine-stimulated cells [4, 5]. Inflammatory transcription factors belong to the intracellular signaling molecules, which may be involved in the refractoriness of macrophages’ responses to repeated stimulation with LPS. In this context, special attention was directed to NFκB. This transcription factor is directly activated within the LPS-TLR-4-stimulated signaling pathway and translocates into the nucleus of LPS-stimulated cells leading to the expression and formation of pro-inflammatory cytokines with TNF-α as initial mediator [46]. However, this function of NFκB depends on its p65 protein subunit, which forms a heterodimer with a p50 protein subunit. One of the suggested mechanisms for the manifestation of LPS tolerance is a replacement of the NFκB heterodimeric form p50-p65 by the homodimeric form p50-p50, which lacks a transactivation domain and exerts an inhibitory influence on inflammatory gene expression [10]. This switch causes a drastic change of the LPS-activated genes, resulting in hyporesponsiveness of TNF-transcription and more pronounced transcription of genes with anti-inflammatory capacities including IL-10 [11, 12, 15, 46, 47]. The enhanced IL-10/TNF-α expression ratios reported here (Figs. 1 and 2) indicate that similar changes occur in primary cultures from rat DRG and SDH primary cultures after induction of LPS tolerance. The antibody, which we employed to quantify the strength of nuclear NFκB immunoreactivity (Fig. 5), is directed against the p65-subunit of NFκB. The reduced nuclear NFκB immunoreactivity, which we determined in the “LPS tolerant” group (LPS1/LPS10 compared to PBS/LPS10) supports the view that a switch from the p50-p65 heterodimer of NFκB to the p50-p50 homodimer may also occur in microglial cells of a given central nervous structure, here the SDH.

STAT3 was the next inflammatory transcription factor, which we investigated with regard to its LPS-induced accumulation in nuclei of cells from the afferent somatosensory system under condition of LPS tolerance. The activation of STAT3 after stimulation with LPS is mediated by a family of LPS-induced cytokines, the “gp130 cytokine receptor family” with IL-6 as its most prominent member [48]. In CNS structures STAT3 seems to participate in various inflammatory responses [49] with astrocytes as a main cellular target for STAT3-activating signal molecules [50, 51]. Thus, activation of astrocytes in the spinal dorsal horn is suggested to play a role in chronic pain states [51]. On the other hand it was reported that inhibition of STAT3 signaling in brain astrocytes reduced experimental brain metastases [52]. In line with these studies, LPS-induced activation of STAT3 could exclusively be shown in astrocytes from SDH primary cultures (Fig. 6). In the context of our study, we were interested in the relative STAT3 expression in GFAP positive astrocytes in our SDH primary culture in the state of LPS tolerance. The mean strength of nuclear STAT3 signals was enhanced after treatment with either a moderate dose or a high dose of LPS (Fig. 6), suggesting that either LPS itself or the pro-inflammatory cytokines released after LPS treatment led to an increased STAT3 activation. In tolerant SDH astrocytes the mean strength of nuclear STAT3 signals decreased, indicating attenuated activation of STAT3. As STAT3 seems to play a role in mediating the anti-inflammatory effects of IL-10 [53], we suggest a fast increase of its activation in astrocytes (2 h) after exposure to LPS and a subsequent decrease after 20 h, which is supported by the work from other groups [54, 55]. The functional relevance of the observed LPS tolerance effect concerning STAT3 activation in astrocytes of the rat SDH still has to be determined. Astrocytes are not present in primary cultures of rat DRG [4]. Interestingly, we detected nuclear STAT3 immunoreactivity in DRG neurons in line with previous studies [4] and an influence of acute stimulation with LPS10, which caused an increase of nuclear STAT3 signals (Fig. 9). The increase of nuclear STAT3 immunoreactivity was abolished in the state of LPS tolerance (Fig. 9). This observation may have functional consequences for the sensitization of primary nociceptive neurons upon inflammatory stimulation. It was reported that inflammatory STAT3 activation in DRG neurons sensitizes peripheral nociceptors via upregulation of TRPV1 [56]. A state of LPS tolerance might therefore have some beneficial effect with regard to the manifestation of inflammatory pain by reducing the inflammation-evoked sensitization of nociceptive neurons. Unfortunately, we could not detect a down-regulation of TRPV1-expression under conditions of LPS tolerance (Fig. 1). However, other studies revealed effects of inflammatory mediators on the TRPV1 channel particularly via posttranslational mechanisms (e.g. phosphorylation) that lead to enhanced membrane availability without observing changes in transcription [57, 58]. For example, phosphorylation of TRPV1 at a single tyrosine residue under the influence of nerve growth factor is followed by insertion of TRPV1 channels into the surface membrane [59]. Thus, the process of sensitization may rather involve enhanced translocation of TRPV1 channels into the membrane of nociceptors than increased mRNA expression [60]. A possible effect of LPS tolerance on sensitization of nociceptive neurons might therefore rather involve an influence of modified intracellular signaling pathways and thereby a modification (reduction) of TRPV1 trafficking to the plasma membrane [58]. Therefore, we cannot exclude the possibility that there is some inhibitory effect on exaggerated nociception in LPS-tolerant DRG neurons despite the lack of an influence on TRPV1 expression. Other experimental approaches are required to evaluate translocation of TRPV1 from the cytosol to the plasma membrane, especially under the influence of LPS tolerance.

Finally, we investigated a third transcription factor, NF-IL6, which has pro-inflammatory properties during the early stage of LPS-induced systemic inflammation, but exerts anti-inflammatory effects during the later stage of the inflammatory process [61]. Here we show (Figs. 7 and 8) that the LPS induced increase of nuclear NF-IL6 immunoreactivity is blunted in the state of LPS tolerance in ED1-positive macrophages (DRG) and microglial cells (SDH). The long-term exposure to the low LPS dose (LPS1) evoked a prolonged increase of nuclear NF-IL6 immunoreactivity, which was still present after a subsequent short-term exposure to the solvent PBS in DRG primary cultures (Fig. 7). This effect was also observed in microglial cells from SDH, although to a smaller degree (Fig. 8). Nevertheless, there was a striking difference between both cultures with regard to nuclear NF-IL6 activation. Our results show a significant decrease in tolerant (LPS1/LPS10) DRG macrophages compared to the LPS1/PBS group. Such an effect was absent in primary cultures from the rat SDH. The reduction of the strength of nuclear NF-IL6 signals in LPS-tolerant DRG macrophages after short-term stimulation with LPS10 can be possibly explained by active down-regulation of NF-IL6 by cytokines distinct from IL-10 [62]. Furthermore, a breakdown of the inflammatory transcription factor NF-IL6 due to LPS tolerance could be another plausible explanation for these results. To investigate whether an active downregulation or a breakdown of the transcription factor NF-IL6 plays a role in the manifestation of LPS tolerance, further experiments, e.g. the use of NF-IL6 knockout mice, would be needed.

As previously reported [4], we also detected nuclear NF-IL6 immunoreactivity in DRG neurons (Fig. 10), which was enhanced in naïve cells by short-term stimulation with LPS. The pre-exposure with LPS1 again caused a long-lasting increase of neuronal NF-IL6 activation. In contrast to macrophages, the LPS1-induced nuclear NF-IL6 activation in neurons was not attenuated by the short-term stimulation with LPS10. In contrast to STAT3 [56, 63], no information from literature is available for a sensitizing effect of NF-IL6 on nociceptive DRG neurons. This question could be addressed by the use of DRG primary cultures from NF-IL6-deficient mice in future studies.

### The impact of LPS tolerance in structures of the peripheral and central nervous system

There are just a few studies, in which the manifestation of LPS tolerance was investigated in structures from the nervous system [22–24, 34]. In these studies, tolerance effects were detected in microglial cells and perivascular macrophages. Our present study on structures of the afferent somatosensory system supports this evidence in so far that the most pronounced tolerance effects were also observed in microglial cells (SDH) and macrophages (DRG). However, we present data showing that LPS tolerance to some degree also develops in astrocytes from the SDH and in neurons from DRG. In the brain, the transient hyporesponsiveness to LPS in the state of tolerance was interpreted as a self-protecting mechanism of the brain against an excessive formation of key mediators of the inflammatory response, for example reactive oxygen species (ROS), nitric oxide via induction of inducible nitric oxide synthase (iNOS) or pro-inflammatory cytokines, such as TNF-α [22–24]. LPS tolerance might thus be a mechanism, which limits the activation of innate immunity within the brain and thereby protects this sensitive tissue from exaggerated immune responses via depressing the activity of transcription factors with pro-inflammatory capacities [23, 64]. Still, there are some indications for modified properties of neurons in the state of LPS tolerance, which still have to be determined with regard to their functional relevance. In some recent studies, a state of LPS tolerance was, indeed shown to have positive effects on stress-induced behavioral abnormalities [65] or on neuropathology and cognitive deficits in model of status epilepticus [66]. Models of LPS-pre-conditioning seem thus to have capacities for protective value in central nervous structures. In the case of neurons from the afferent somatosensory systems it will, for example, be of interest to investigate, whether inflammation related changes of neuronal responses can be modified in a state of LPS tolerance or generally in states of immune tolerance.
